# Response of *Candida albicans* white and opaque cells to phagocytosis by macrophages suggests that opaque cells are “pre-adapted”

**DOI:** 10.1128/msphere.00690-25

**Published:** 2025-12-18

**Authors:** Matthew B. Lohse, Megan E. Garber, Haley Gause, Jenny Y. Zhang, Anika Ramachandran, Carrie E. Graham, Alexander D. Johnson

**Affiliations:** 1 Department of Microbiology and Immunology, University of California – San Francisco 8785, San Francisco, California, USA; 2 Tetrad Graduate Program, University of California – San Francisco 8785, San Francisco, California, USA; University of Guelph, Guelph, Ontario, Canada

**Keywords:** *Candida albicans*, white-opaque switching, host-pathogen interactions, fungal pathogen, response to macrophage, *Candida albicans*-innate immune system interactions

## Abstract

*Candida albicans* is a normal resident of the human gut and mucosal microbiomes and also an opportunistic fungal pathogen. It undergoes several morphological transitions, one of which is white-opaque switching, where *C. albicans* reversibly alternates between two distinct cell types, namely, “white” and “opaque.” Each state, which is maintained by a complex transcriptional feedback loop, is heritable through many cell divisions. To date, most research works on interactions between *C. albicans* and the innate immune system have utilized white cells. In this paper, we examine the response of opaque cells following phagocytosis by murine macrophage cell lines and compare it to the response of white cells. White cells are known to rapidly form hyphae that can rupture macrophages, but we show here that opaque cells continue to proliferate as yeast-form opaque cells within the macrophage. Before phagocytosis, white and opaque cells differ markedly in the mRNAs they express and therefore enter macrophages as two distinct types of cells. We were surprised to observe that, within macrophages, the transcriptional profiles of white and opaque cells became much more similar to each other. This convergence was driven, in part, by the upregulation, in white cells, of a set of genes that were already expressed in opaque cells prior to macrophage exposure. These observations indicate that opaque cells, compared to white cells, are “pre-adapted” for life within host macrophages.

**IMPORTANCE:**

The human fungal pathogen *Candida albicans* undergoes several morphological transitions, one of which is white-opaque switching. Although most research works on interactions between *C. albicans* and the innate immune system have focused on white cells, opaque cells have been shown to interact with macrophages differently compared to white cells. In this study, we examine the transcriptional response of opaque cells to phagocytosis and compare it to that of white cells. Despite differences in how the two cell types proliferate following phagocytosis, their transcriptional responses strongly overlap, and fewer genes are differentially expressed between white and opaque cells following phagocytosis than observed in media lacking macrophages. Unexpectedly, the responses of both white and opaque cells favor genes that were already upregulated in opaque cells (relative to white cells) before exposure to macrophages; these observations suggest that opaque cells are “pre-adapted” for life within macrophages.

## INTRODUCTION

The opportunistic fungal pathogen *Candida albicans* is a common component of a healthy human microbiome. Although normally asymptomatic, *C. albicans* can cause systemic bloodstream infections with mortality rates that exceed 40% if the host’s immune system is compromised (1–11). Much of the research on *C. albicans* has been focused on its ability to switch between several distinct morphologies. One such system is white-opaque switching, where *C. albicans* alternates between two cell types, namely, “white” and “opaque,” which exhibit distinct cellular and colony morphologies (12–17). Each state is stable across many cell divisions; switching between these two cell types occurs stochastically approximately once every 10^4^ cell divisions under standard laboratory conditions, is reversible, occurs without any chromosomal rearrangements or DNA sequence changes, and relies on stable transcription feedback loops (18–20). Although switching occurs stochastically, environmental signals, such as elevated temperature or exposure to N-acetylglucosamine (GlcNAc), can drive switching from one cell type to the other (12, 18, 21, 22). Approximately one-sixth of the *C. albicans* genome is differentially expressed between the two cell types, resulting in a number of characteristics specific to one or the other cell type (23, 24). For example, they express different metabolic enzymes, resulting in distinct metabolic preferences which, in turn, affect growth rates and the ability to proliferate on different nutrients (22, 23, 25, 26). The two cell types also respond differently to specific environmental cues; for example, opaque but not white cells form hyphae in response to low levels of nitrogen or phosphate (22, 27–30). Finally, the two cell types show drastic differences in their ability to undergo a parasexual cycle, with opaque cells mating approximately one million times better than white cells (31).

The interactions of *C. albicans* white cells with the innate immune system have been extensively studied. Phagocytosis of *C. albicans* by macrophages and neutrophils is largely mediated by Dectin-1 binding to β−1,3-glucans in the *C. albicans* cell wall, with the level of surface-exposed β−1,3-glucans determining the extent of Dectin-1 binding (32–36). Once phagocytosed, *C. albicans* white cells begin to form hyphae and upregulate a number of cell wall- and hyphal-related genes as well as genes associated with the import and metabolism of alternative carbon sources, including amino acids, fatty acids, organic acids, and lipids, likely in response to the glucose-poor nature of the phagosome (37–41). Elements of this transcriptional response are conserved across a set of six different *Candida* species regardless of whether these species are pathogenic or able to form hyphae, suggesting that this response is ancient (41). In the few cases where they have been studied, opaque cells have been shown to interact with the host and host innate immune system in a manner distinct from white cells. For example, compared to white cells, opaque cells are phagocytosed at a significantly reduced level by neutrophils and macrophages, suggesting that switching from white to opaque cells may help *C. albicans* evade the innate immune system (42–44). The two cell types also differ in their abilities to colonize specific niches in the host; although white cells significantly outcompete opaque cells in some organs (e.g., the kidney), in other organs (e.g., heart and brain), colonization by the two cell types is roughly equivalent (45–48).

The majority of research on *C. albicans* has been carried out in SC5314, an a/α strain that is incapable of white-opaque switching unless it is converted to an “a” or “α” strain by genetic manipulation. Because SC5314 did not exhibit switching, the interactions of opaque cells with the innate immune system did not initially seem to be of high priority. It was subsequently discovered, however, that SC5314 was not representative of the majority of *C. albicans* clinical isolates: up to two-thirds of clinical *C. albicans* a/α isolates are capable of forming opaque cells without acquiring mutations (49–52). Given the potential importance of opaque cells in the host, we examined the opaque cell response to phagocytosis by murine macrophages and showed that, following phagocytosis, the response of opaque cells is markedly different from that of white cells. In contrast to white cells, which form hyphae and rupture macrophages, opaque cells continue to divide in macrophages as yeast-form cells. From a series of RNA-seq experiments, we found that, despite these morphological differences, the transcriptional profile of white and opaque cells in macrophages became more similar to each other after phagocytosis than before; in other words, fewer genes were differentially expressed between the two cell types within macrophages than before phagocytosis. This pattern occurs, in part, because a number of genes induced by white cells in response to phagocytosis were already expressed in opaque cells before phagocytosis. These results show that, in addition to their lower phagocytosis rate, opaque cells differ from white cells in being transcriptionally “pre-adapted” for phagocytosis. We suggest that this pre-adaptation contributes to the specialized response of opaque cells—proliferation instead of hyphal formation leading to rapid lysis—to phagocytosis.

## RESULTS

### Opaque cells proliferate as yeast, not hyphae, in the macrophage

**Fig 1:**
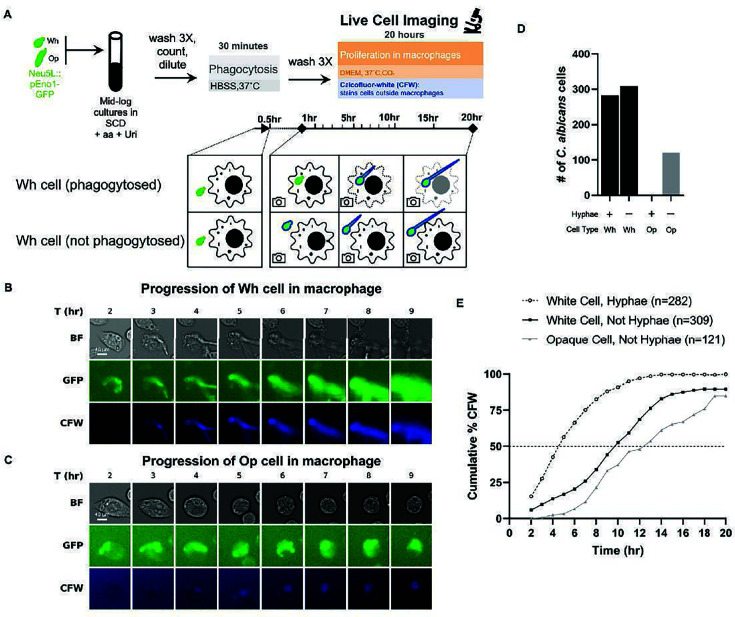
White and opaque *C. albicans* cells proliferate differently following phagocytosis by macrophages. (**A**) Overview of live-cell imaging using white or opaque cells that are constitutively expressing GFP (details provided in Materials and Methods). Representative images from live-cell imaging for (**B**) white or (**C**) opaque cells, showing the time frame from the 2- to 9-h time points for each image. The Brightfield (BF), GFP channel (constitutively expressed by *C. albicans* cells), and CFW (cells exposed to media due to not being phagocytosed or escaping from the macrophage) channels are shown for each image. (**D**) Distribution of hyphal formation, or lack thereof, in phagocytosed white (black) and opaque (gray) cells. (**E**) The cumulative percentage of cells that have escaped from the macrophage at each time point, as indicated by CFW staining. The white cell data are separated by whether or not the phagocytosed white cells formed hyphae. Results in panels **D** and **E** represent events from experiments with an MOI of 1 that meet the exclusion criteria, as described in the Materials and Methods.

To compare the behavior of white and opaque cells in the macrophage, we performed live-cell imaging for 20 h following phagocytosis by the J774A.1 murine macrophage cell line (Fig. 1A). To minimize the number of nonphagocytosed cells and reduce hyphae formation prior to phagocytosis (53), *C. albicans* was added at a multiplicity of infection (MOI) of 1 to macrophages in Hanks’ Balanced Salt Solution (HBSS) lacking fetal bovine serum (FBS) for 30 min at 37°C (with ambient CO_2_) prior to the addition of Dulbecco’s modified Eagle’s medium (DMEM) with FBS and 5% CO_2_ (53). We used *C. albicans* cells that constitutively expressed GFP from the *ENO1* promoter at the neutral locus (NEU5L) to allow for easy visualization. The macrophage-impermeable dye Calcofluor-white (CFW) was included with the DMEM to stain unphagocytosed cells as well as cells that escaped from the macrophage (54). Consistent with previous reports, approximately half of the phagocytosed white cells quickly began to form hyphae under these conditions (Fig. 1B and D; File S1). Using the time it took for half of the starting cells to be stained by CFW as an indicator of escape, hyphae-forming white cells escaped from the macrophage after approximately 5 h, while the yeast-form white cells escaped after 10 h (Fig. 1E; File S1). In contrast, phagocytosed opaque cells did not form hyphae (98%, 121 of 123 macrophages tracked) but instead continued to visibly proliferate as yeast-form opaque cells, frequently undergoing multiple rounds of cell division before eventually escaping the macrophage after 13 h (Fig. 1C, D and E; File S1). We note that differences in the MOI or the number of cells initially present in the macrophage (one versus two cells) did not affect the aforementioned trends (Fig. S1; File S1). The persistence of yeast-form opaque cells in the macrophage for this length of time is especially notable because exposure to elevated temperature (37°C) in a wide variety of lab media results in *en masse* opaque-to-white switching after approximately 4 to 6 h (12, 18, 55). Thus, the stability of opaque cells in the macrophage represents a rare case where the opaque cell program is actively maintained and propagated at elevated temperatures.

### Phagocytosis assay optimization for transcriptional profiling of opaque cells

**Fig 2:**
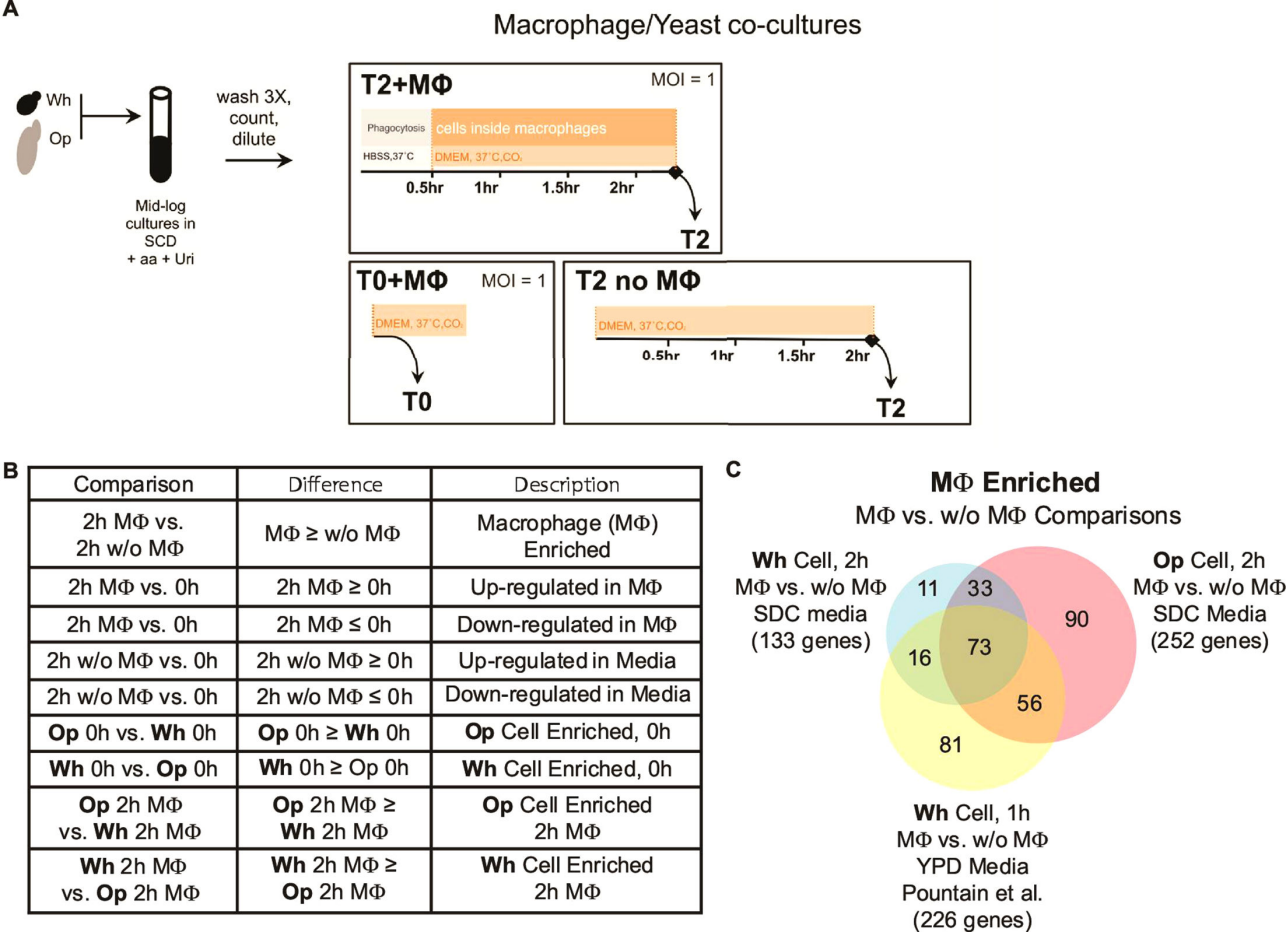
The transcriptional response to phagocytosis is largely conserved between white and opaque cells. (**A**) Overview of the experimental strategy for the RNA-seq experiment (details provided in Materials and Methods). White or opaque cells were cultured in SCD + aa + uri to mid-log phase such that they were actively growing at the time of the experiment. To prepare the cells for the subsequent step, cells were washed three times, counted, and diluted to the same concentration such that the same number of cells were used between all tests and replicates. The prepared cells were then added at the same cell number/MOI to three tests. For the first test (top box), cells were added at an MOI of 1 to tissue culture plates containing macrophages (MΦ) in HBSS. After 30 min at 37˚C without CO_2_, this co-culture was washed three times with HBSS, the media was replaced with DMEM, and the co-cultures were then incubated at 37˚C with 5% CO_2_ for 2 h, at which point they were harvested. For the second test (bottom left box), cells were added at an MOI of 1 to tissue culture plates without MΦ in DMEM at 37˚C with CO_2_ and then were immediately harvested. For the third test (bottom right box), cells were added at an MOI of 1 to tissue culture plates without MΦ in DMEM. The *C. albicans* monocultures were then incubated at 37˚C with 5% CO_2_ for 2 h at which point they were harvested. (**B**) Guide to the comparisons between data sets discussed in this paper and how differences between specific samples are commonly described. (**C**) Venn diagram of *C. albicans* genes that were upregulated at least fourfold in phagocytosed cells relative to un-phagocytosed cells (MΦ up, 2 h MΦ vs 2 h w/o MΦ) in opaque or white cells from this study or in the 1 h white cell data set reported by Pountain and colleagues (41).

To further explore the differences between the white and opaque cell responses, we transcriptionally profiled white and opaque cells that had been phagocytosed by J774A.1 murine macrophages. Although a full description of the experimental setup is presented in the Materials and Methods section, we briefly note several experimental details intended to ensure that the opaque cell cultures did not contain an appreciable fraction of white cells that could confound the analysis. All *C. albicans* pre-culturing was conducted on SCD +aa + Uri at 25°C to minimize opaque-to-white switching before exposure to macrophages. Cell-type homogeneity of all cultures was verified by microscopy prior to incubation with macrophages. Following the previously described 30 min HBSS phagocytosis step, cells were inoculated in DMEM with FBS at 37°C with 5% CO_2_ for 2 h before harvesting (Fig. 2A). The 2 h incubation window represented a balance between allowing adequate time for opaque cell phagocytosis while minimizing white cell escape from macrophages.

Each pre-culture was divided to produce three distinct samples: (i) one harvested immediately (within 1 to 2 min) after adding to wells that contain DMEM and macrophages (“0 h”), (ii) one harvested after 2 h incubated in DMEM with macrophages present (“2 h MΦ”), and (iii) one harvested after 2 h incubated in DMEM without macrophages present (“2 h w/o MΦ”). We note that sample 3 did not undergo the HBSS phagocytosis and wash steps that sample 2 did. Aliquots were taken from each sample of each culture immediately after harvesting and plated to monitor the proportion of white and opaque cells in each sample.

No cell-type switching was observed for any of the white cell samples or the 0 h opaque cell samples, while low levels of opaque-to-white switching were observed in the opaque 2 h MΦ and 2 h w/o MΦ samples (Table S1). These levels of “contaminating” white cells are sufficiently low to have no consequences for the interpretation of the RNA-seq data.

Principal component analysis (PCA) confirms the baseline expectations of the experiment. First, the largest source of variance (accounting for 49%) is the response of *C. albicans* to environmental differences (temperature, CO_2_, and serum) between the 0 h and 2 h samples, irrespective of the presence of macrophages (Fig. S2A). This observation is consistent with that of a large literature documenting changes in *C. albicans* gene expression elicited by changes in these environmental factors. The second principal component (accounting for 27% of variance) is due to other differences, including cell type and macrophage effects (Fig. S2A). We next analyze the transcriptional changes underlying this second principal component.

### White and opaque cells have a similar transcriptional profile during phagocytosis

Unless otherwise noted, the RNA-seq analyses presented below focus on genes whose expression changes were large (at least fourfold between samples) and that have a P_adj_ less than 0.05. These thresholds were chosen to highlight patterns that are derived from high-magnitude differences and to minimize the influence of the many small, likely indirect, changes. We first consider the transcriptional changes that were observed in the presence of macrophages relative to the absence of macrophages (2 h MΦ vs 2 h w/o MΦ) at the same time point, 2 h, in the same medium at the same temperature (e.g., DMEM media supplemented with FBS at 37°C, 5% CO_2_) (Fig. 2B). Genes that were expressed at higher levels in macrophages compared with the medium alone are denoted as “macrophage-enriched.” We observed 133 genes that were “macrophage-enriched” in white cells, and these genes were largely encompassed by the 252 “macrophage-enriched” genes that we observed in opaque cells (106 shared genes, *P* < 1e-15, Fig. 2C). The “macrophage-enriched” genes in both our white and opaque cell data sets exhibit significant overlap with the *C. albicans* a/α white cell “macrophage-enriched” set previously reported by Pountain and colleagues (41). Specifically, two-thirds of our white cell “macrophage-enriched” set (89 of 133 genes, *P* < 1e-15, Fig. 2C) and half of our opaque cell “macrophage-enriched” gene set (129 of 252 genes, *P* < 1e-15, Fig. 2C) overlap with the 226 genes that were “macrophage-enriched” in the Pountain data set. This high degree of overlap occurs despite the numerous experimental differences between the two studies (e.g., different preculture conditions, different *C. albicans* strains, a 2 h versus a 1 h phagocytosis step, and use of the J774A.1 macrophage cell line rather than bone marrow-derived macrophages). Also consistent with previous studies (37–41), the induction of genes associated with the utilization of alternative (non-glucose) carbon sources was prominent in our “macrophage-enriched” genes in both cell types. These include genes involved in carbohydrate metabolism (e.g., the pyruvate, glyoxylate, dicarboxylate, and propanoate KEGG modules), lipid metabolism (e.g., fatty acid degradation and genes localized to the peroxisome), amino acid biosynthesis or degradation (e.g., valine, leucine, and isoleucine degradation, beta-alanine metabolism, and glutathione metabolism), and a wide range of transporters.

### “Macrophage-enriched” genes include those upregulated in macrophages but also those downregulated in media alone

**Fig 3:**
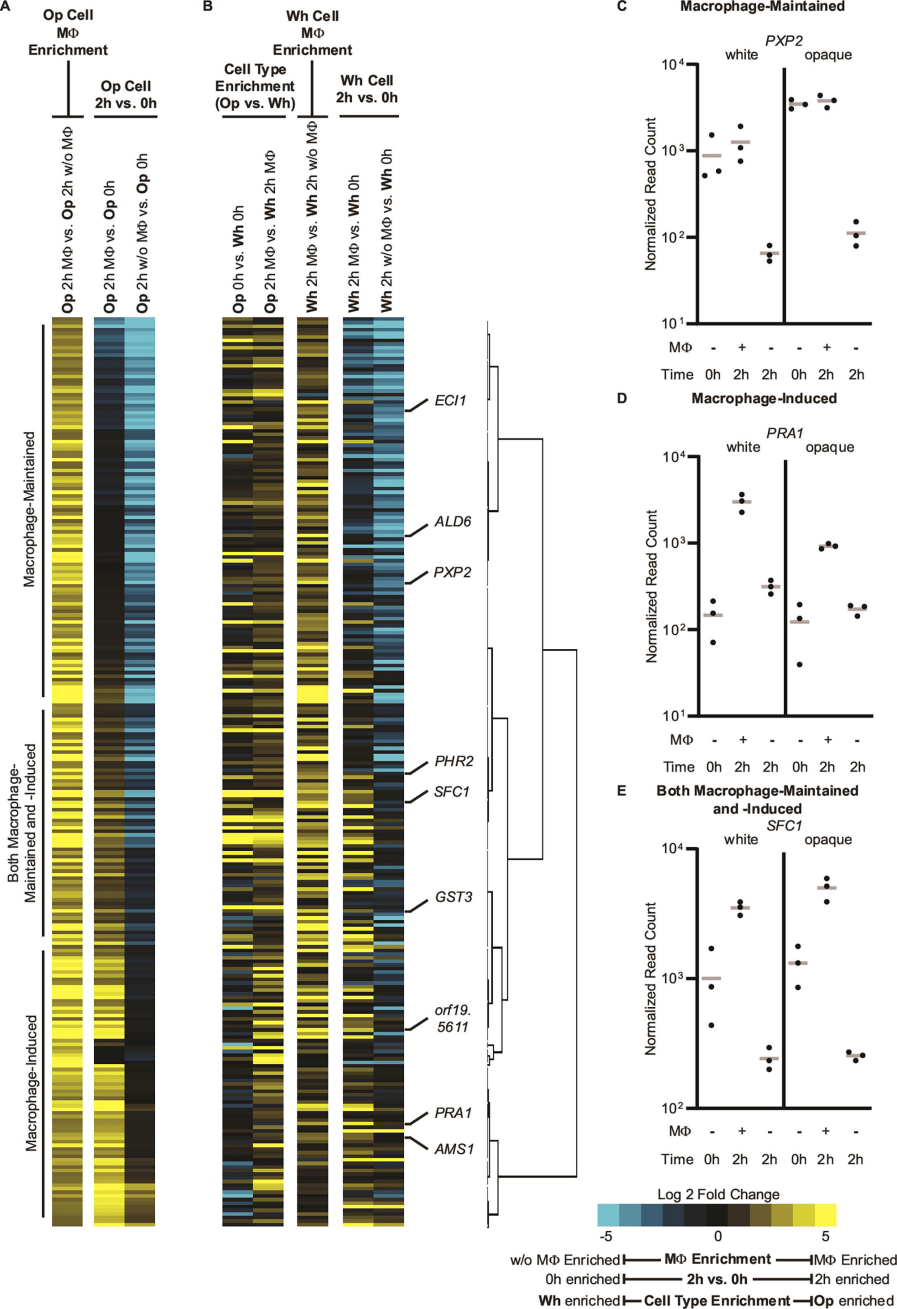
The “macrophage-enriched” response to phagocytosis (MΦ up, 2 h MΦ vs 2 h w/o MΦ) involves a mixture of upregulating genes in macrophages (2 h MΦ vs 0 h, “macrophage-induced”) and downregulating genes when macrophages are not present (2 h w/o MΦ vs 0 h, “macrophage-maintained”). (**A–B**) Heatmap of the 252 genes that are at least fourfold macrophage-enriched in opaque cells. The underlying causes for the macrophage enrichment (e.g., “macrophage-maintained”) and positions of the genes from Panels **C–E** and Fig. S3A are indicated. (**A**) Gene expression profiles in opaque cells. (**B**) Gene expression profiles in white cells and cell-type enrichment profiles. Equivalent heatmaps for genes that are macrophage-enriched in white cells are provided in Fig. S2. (**C–E**) Normalized read counts for (**C**) macrophage-maintained *PXP2*; (**D**) macrophage-induced *PRA1*; (**E**) both macrophage-maintained and -induced *SFC1* are shown for each sample in each cell type. Points represent the normalized read count for each of the three repeats, and the gray line indicates the mean. Read counts for additional genes in each category are provided in Fig. S3A.

Two possibilities could account for those genes scored as “macrophage-enriched,” as described below. These genes could be upregulated in macrophages compared to the 0 h time point (2 h MΦ vs 0 h) or they could be downregulated in the medium alone (2 h w/o MΦ vs 0 h), but not in macrophages, at the 2 h time point (in other words, they could be downregulated due to environmental effects) (Fig. 2B). To distinguish between these possibilities, we incorporated the “0 h” timepoint RNA-seq data in our analysis. We found that both possibilities occur. One set of “macrophage-enriched” genes, which we term “macrophage-maintained,” are genes that are expressed at the 0 h timepoint, maintained at the 2 h timepoint in macrophages, but downregulated in the 2 h media only samples. Of 133 white cell and 252 opaque cell “macrophage-enriched” genes, approximately one-third fall into this “macrophage-maintained” category (Fig. 3A through C; Fig. S2 and S3). Another third are induced in macrophages compared to the 0 h time point and will be referred to as “macrophage-induced” genes (Fig. 3A and B; Fig. S2 and S3). The remaining one-third of the “macrophage-enriched” genes are subject to both of the aforementioned effects (Fig. 3A, B and E; Fig. S2 and S3).

All three groups contain transporters and cell wall-/cell surface-associated genes. The “macrophage-maintained” genes are over-represented for amino acid pathways (e.g., valine, leucine, and isoleucine degradation) as well as fatty acid degradation and assorted carbohydrate metabolic KEGG modules (e.g., glyoxylate, pyruvate, and glycolysis-gluconeogenesis). This analysis indicates that a large portion of what has been traditionally perceived as the response of *C. albicans* to macrophages are genes that are well-expressed in *Candida* before encountering macrophages and whose expression is maintained in macrophages; in a sense, macrophages prevent their downregulation compared to media only, but do not induce the expressions of these genes.

### Phagocytosis induces significant changes in genes differentially expressed between white and opaque cells

**Fig 4:**
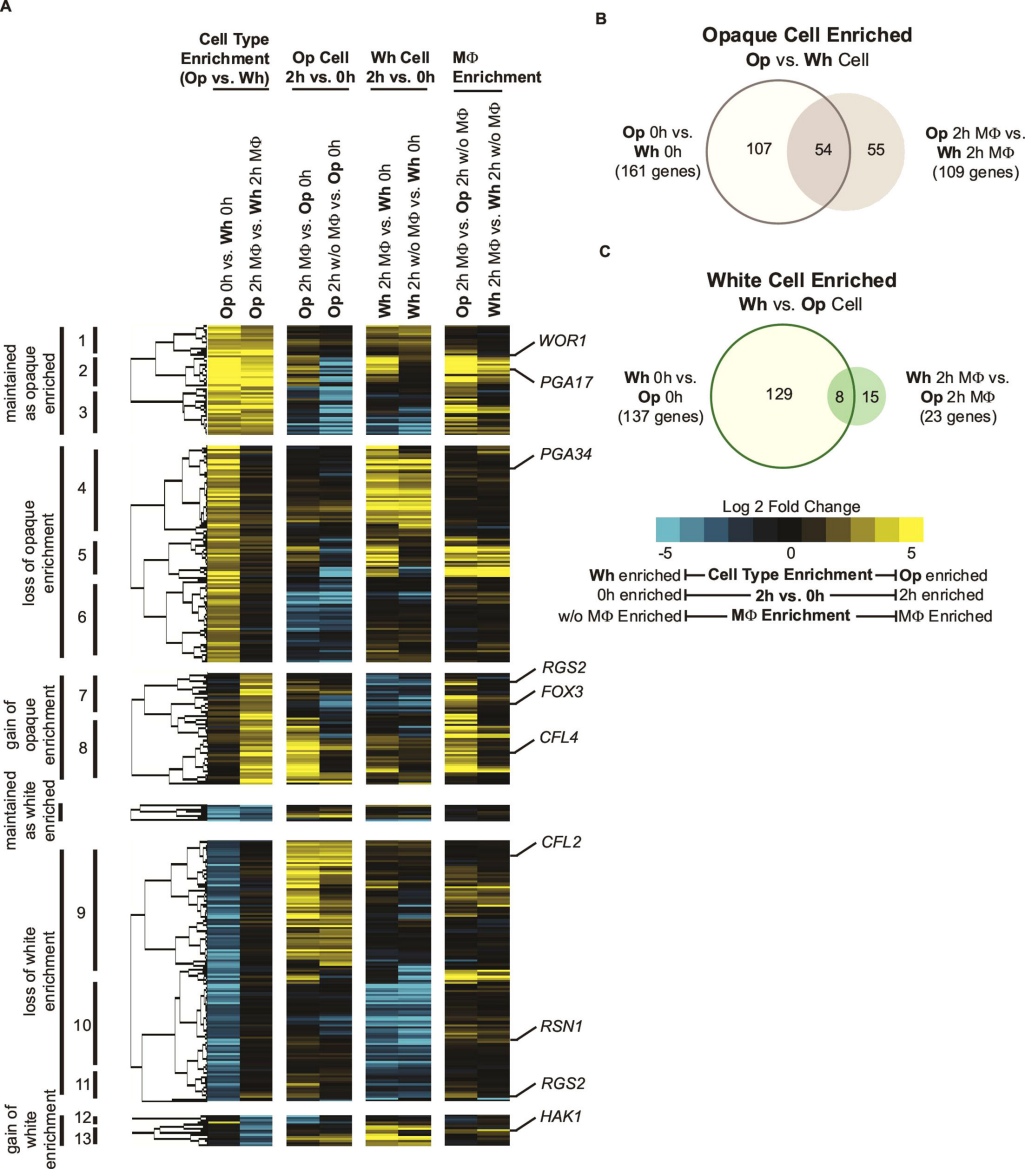
Unlike the white cell type-enriched gene set, the opaque cell type-enriched gene set persists and gains new genes following phagocytosis. (**A**) Heatmap of the 366 genes that are at least fourfold opaque or white cell type-enriched at either 0 h or after 2 h in the presence of macrophages. Genes are separated by whether cell type-enrichment was maintained (0 h and 2 h MΦ), lost (0 h only), or gained (2 h MΦ only). The positions of the opaque-and white-enriched genes from Fig. S4 are indicated to the right of the heatmap. Genes that are maintained as opaque-enriched can be divided into those that are not changing (group 1), that are upregulated in both cell types (group 2), and those that are downregulated in both cell types (group 3). Genes where opaque cell-type enrichment is lost can be divided into those that are upregulated in white cells (group 4), upregulated to a greater extent in white cells (group 5), and that are downregulated in opaque cells (group 6). Genes where opaque cell type enrichment is gained can be divided into those that are down-regulated in white cells (group 7) and that are up-regulated in opaque cells (group 8). Genes where white cell-type enrichment is lost can be divided into those that are upregulated in opaque cells (group 9), that are downregulated in white cells (group 10), and those that are both upregulated in opaque cells and downregulated in white cells (group 11). Genes where white cell-type enrichment is gained can be divided into those that are downregulated in opaque cells (group 12) and that are upregulated in white cells (group 13). (**B–C**) Venn diagrams showing the overlap in genes enriched at least fourfold in (**B**) opaque cells or (**C**) white cells in the 0 h and/or 2 h MΦ samples.

We now consider the differences in the white and opaque cell responses to phagocytosis. At the 0 h time point, there are 161 opaque-enriched genes, that is, genes expressed at least fourfold higher in opaque cells compared to white cells (Fig. 2B). After 2 h in the presence of macrophages, only 54 of these genes are still opaque-enriched (Fig. 4A and B). Most of the genes that are opaque-enriched at 0 h but not after 2 h of exposure to macrophages (70 of 107 genes) are the results of these genes being upregulated in white cells exposed to macrophages at the 2-h time point, thereby neutralizing their opaque-specificity (Fig. S4A). The levels of white-enriched genes also diminish during phagocytosis. Of the 137 white-enriched genes at the 0 h timepoint (that is genes expressed at least fourfold higher in white cells compared to opaque cells [Fig. 2B]), almost all (129) are no longer white-enriched at the 2 h timepoint after exposure to macrophages (Fig. 4A and C). This loss is due to three types of expression changes: 48 genes that were white-enriched at 0 h are downregulated by white cells in macrophages (Fig. 4A; Fig. S4B), 67 genes that were white-enriched at 0 h are upregulated by opaque cells in macrophages (Fig. 4A; Fig. S4C), and 13 genes that were white-enriched at 0 h are subject to both effects (Fig. 4A; Fig. S4D). A much smaller set of genes become white- or opaque-enriched only after exposure to macrophages. For example, 11 genes are specifically upregulated in white cells after exposure to macrophages and become white-enriched at the 2 h time point (Fig. 4A; Fig. S4E). Likewise, 38 genes are specifically upregulated in opaque cells after exposure to macrophages and become opaque-enriched at the 2 h time point (Fig. 4A; Fig. S4F). Considering all of the aforementioned transcriptional changes, fewer than half as many genes are differentially expressed between the two cell types in macrophages than are at 0 h in standard laboratory media (132 versus 298 genes); this trend is robust across a range of fold changes and P_adj_ thresholds (File S2). In other words, the gene expression profiles of white and opaque cells are more similar to each other after exposure to macrophages (2 h MΦ) than before (0 h).

### Opaque cells appear “pre-adapted” to the macrophage compared to white cells

**Fig 5:**
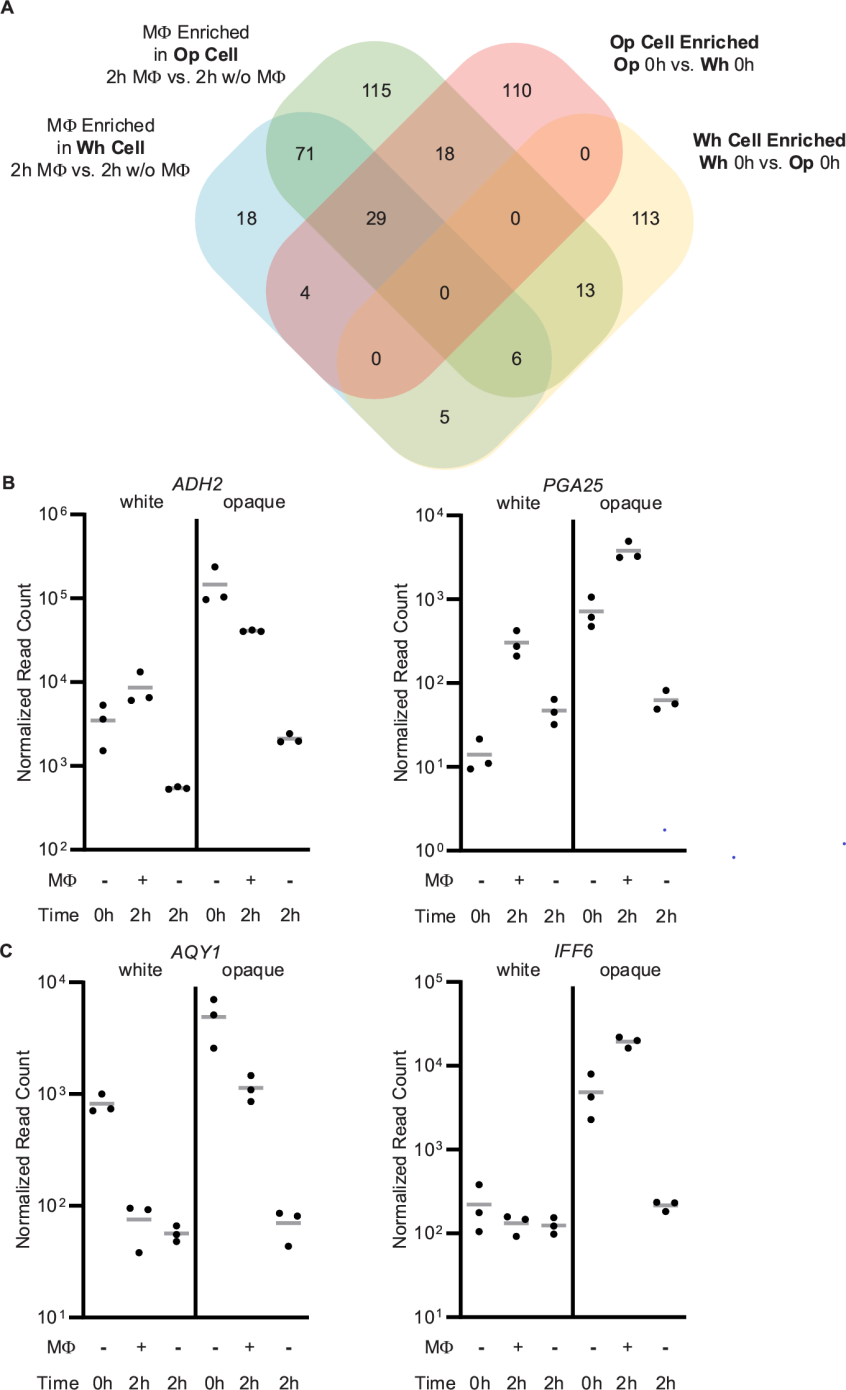
The opaque cell type-enriched gene sets significantly overlap with the macrophage-enriched gene sets from both white and opaque cells. (**A**) Comparison of the genes that are at least fourfold macrophage-enriched (2 h MΦ vs 2 h w/o MΦ) in white and/or opaque cells with the genes that are either fourfold white cell type- or opaque cell type-enriched in the 0 h samples. (**B**) Normalized read counts for *ADH2* and *PGA25*, two of the 29 white and opaque macrophage-enriched genes that are also opaque cell enriched at 0 h. (**C**) Normalized read counts for *AQY1* and *IFF6*, two of the 18 opaque but not white macrophage-enriched genes that are also opaque cell-enriched at 0 h. Points represent the normalized read count for each of the three repeats, and the gray line indicates the mean. Read counts for additional genes are provided in Fig. S5.

As described above, white and opaque cells differ significantly in their gene expression patterns before encountering macrophages. However, following phagocytosis, these gene expression patterns become much more similar to each other. Are these cells more closely related to white or opaque cells? We found that the opaque cell “macrophage-enriched” gene set is more over-represented for genes that are opaque-enriched than white-enriched at the 0 h time point (19 white-enriched genes versus 47 opaque-enriched genes, *P* = 9.8e-7 versus *P* < 1e-15) (Fig. 5; Fig. S5). Similarly, the white cell “macrophage-enriched” gene set is more over-represented for genes that are opaque-enriched than white-enriched at the 0 h time point (11 white-enriched genes versus 33 opaque-enriched genes, *P* = 4.9e-5 versus *P* < 1e-15) (Fig. 5; Fig. S5). In other words, both the white and opaque cell transcriptional responses to macrophages are biased toward a group of genes that were already expressed at higher levels in opaque cells prior to encountering macrophages. This trend suggests that opaque cells are “pre-adapted” for macrophages compared to white cells because, even before they encounter macrophages, they express a significantly larger set of what will become the “macrophage-enriched” genes. Superimposed on this trend are a large number of “macrophage-enriched” genes that are not expressed in a cell type-specific manner at the 0 h time point; in other words, the “preadaptation” of opaque cells represents only a portion of the total response to macrophages.

## DISCUSSION

Previous studies showed that *C. albicans* opaque cells are phagocytosed by murine macrophages at lower levels than white cells (42–44). In this study, we used a combination of time-lapse microscopy and whole-genome transcriptional profiling to compare how white and opaque *C. albicans* cells behave after phagocytosis. The most significant findings of this study are as follows:

First, white and opaque cells behave differently in response to phagocytosis: phagocytosed white cells begin to form elongated hyphae while opaque cells continue to proliferate as yeast-form opaque cells. Elevated temperatures (37°C) normally switch opaque cells to white cells, but in the macrophage at 37°C, opaque cells remain stable and continue to proliferate as opaque cells. Thus, the environment of macrophages actively preserves the opaque state, overriding a signal (37°C) that normally induces switching from the opaque to the white cell types.

Second, the white and opaque cell transcriptional programs are very different before encountering macrophages but become more similar to each other following phagocytosis.

Third, the “macrophage-enriched” response of both white and opaque cells is biased toward a group of genes that were already upregulated in opaque cells (relative to white cells) before exposure to macrophages. In that sense, opaque cells appear prepared (“pre-adapted”) for the macrophage environment.

Fourth, during the course of this work, we determined that genes traditionally scored as “macrophage-enriched” (defined as higher in macrophages at a specific time point compared with “no-macrophage” controls collected at that same time point) show three distinct types of gene expression changes. Many genes are scored as “macrophage-enriched,” not because their expression is upregulated in macrophages, but because they are repressed at the 2 h time point in the no-macrophage medium control. In other words, many of these genes were already expressed at the 0 h time point and remained expressed at similar levels at the 2 h MΦ time point. In a sense, the macrophage environment could be said to prevent these genes from being repressed, but it does not truly induce them. We refer to this class of genes (approximately one-third of macrophage-enriched genes) as “macrophage-maintained.” Another one-third are actively induced in macrophages compared to the 0 h time point (denoted “macrophage-induced”), and the remaining third are subject to both influences.

As described in this paper, white and opaque cells converge on similar expression profiles in macrophages, despite displaying markedly different transcriptional profiles before phagocytosis. How and why might this occur? White-opaque switching is a recently evolved phenotype observed only in *C. albicans* and the two closely related species *Candida dubliniensis* and *Candida tropicalis* (56, 57). In contrast, the transcriptional response of *C. albicans* white cells to phagocytosis has many elements (e.g., induction of genes related to carbon starvation) that are broadly conserved across at least five other *Candida* species (41), three of which (*Candida parapsilosis*, *Lodderomyces elongisporus*, and *Clavispora lusitaniae*) appear to have diverged from a common ancestor before white-opaque switching arose. We propose that opaque cells may have co-opted portions of this conserved macrophage response simply by relaxing the requirement for the presence of macrophages in order for *Candida* to express these genes. In other words, a set of macrophage-response genes is now constitutively expressed in opaque cells. This idea is supported by previous observations that the opaque-cell transcriptional program is heavily focused on the utilization of alternative carbon sources (22, 23).

If the transcriptional response to phagocytosis predates the opaque cell type, why do opaque cells behave so differently from white cells in the macrophage? It has been previously shown that white and opaque cells frequently exhibit different responses to the same environmental signals. For example, conditions that induce hyphae formation in white cells (e.g., serum, high temperature, and nutrient starvation [22, 23, 26]) do not induce hyphae formation in opaque cells, while conditions that induce hyphae formation in opaque cells (e.g., low phosphate, low nitrogen, sorbitol, and GlcNAc) do not induce hyphae formation in white cells (22, 27). Therefore, it is plausible that, due to their gene expression profile established before encountering macrophages, opaque cells avoid hyphae formation inside the macrophage simply because they do not respond to the “white cell-specific” signals that are present in the macrophage. There are several hypotheses that can account for this failure to respond. For example, opaque cells could simply fail to detect the signal(s) that trigger hyphae formation in white cells. Another possibility is that opaque cells detect the signal(s), but hyphal formation is repressed by the opaque cell transcriptional program.

Despite the convergence of the white and opaque cell gene expression patterns in the macrophage, there are some genes whose expression remains cell type-specific, predominantly opaque-specific. The expression of these genes arises from four types of changes: (i) preexisting opaque-enriched genes (compared to white cells) that remain expressed at similar levels in the macrophage and that were not upregulated in white cells (e.g., *WOR1*, Fig. 4A; Fig. S4G), (ii) opaque-enriched genes that were upregulated by approximately the same extent in both opaque and white cells (e.g., *PGA17*, Fig. 4A; Fig. S4H), (iii) genes newly upregulated in the macrophage but only in opaque cells (e.g., *CFL4*, Fig. 4A and F), and (iv) genes that are downregulated in white cells but that remain expressed in opaque cells (e.g., *FOX3*, Fig. 4A; Fig. S4I). We propose that this core program of opaque-enriched genes ensures that opaque cells remain as yeast-form cells for many hours in the macrophage, long after the first wave of gene expression changes has occurred.

Finally, our results suggest that opaque cells, compared to white cells, are prepared for proliferation within the macrophage. Although opaque cells have traditionally been perceived as unstable at the elevated temperatures encountered in the host, the work described here shows that, inside macrophages, opaque cells remain opaque and continue to proliferate as opaque cells despite being held at 37°C. These observations support and extend the idea that opaque cells are well-suited for certain niches within the host and behave differently from white cells. Relative to white cells, opaque cells are less prone to being phagocytosed, are better able to use proteins as a nitrogen source, and have distinct organ/tissue colonization preferences. As shown here, opaque cells stably express “macrophage-enriched” genes before encountering macrophages and proliferate in the macrophage at 37°C for several generations without lysing the macrophage (13–17, 25, 42–48, 58, 59). Although these results do not necessarily mean that opaque cells stably “live” in host macrophages for long periods of time, they indicate that opaque cells are specialized for this niche. Like other pathogens, *C. albicans* has the innate ability to proliferate inside macrophages, but this is only realized in a specialized cell type.

## MATERIALS AND METHODS

### Plasmid and strain construction

Lists of strains, plasmids, and oligonucleotides used in this study can be found in File S3.

The MTL deletion plasmid pMBL745, a recyclable *SAT1*-based equivalent to the previously published Arg4-based pJD1 (60) and nonrecyclable *SAT1*-based pMBL744 (61), was constructed as follows. The 500 bp 5′ and 3′ of the *C. albicans* MTL locus were PCR-amplified, fused together with XhoI and NotI restriction sites positioned between them, and integrated between the SphI and AatII sites of pUC19 to form the previously reported pMBL743 (61). During the pMBL743 construction process, two XmaI sites were added just inside the SphI and AatII sites to allow for easier linearization of the final construct. The recyclable version of the *SAT1* marker from pSFS2a (62) was digested from pSFS2a using XhoI and NotI, purified via gel extraction, and then integrated into pMBL743 between the XhoI and NotI sites to form pMBL745. The resulting plasmid was then linearized by XmaI digestion prior to transformation into *C. albicans*.

The plasmid for constitutively expressed GFP was constructed as follows. The 550 bp *NEUT5L* locus was chosen as the integration site due to the low fitness cost associated with exogenous DNA expression (63). The mid-point of *NEUT5L* is defined here as 1,959 bp downstream of the *GDS1* start codon. Three hundred base pairs downstream of the *NEUT5L* mid-point (3′ *NEUT5L* homology) were PCR-amplified with a 5′ NotI site and a 3′ AatII site. Following digestion with NotI and AatII, this fragment was ligated into the similarly digested GFP-containing *SAT1* flipper cassette of pMBL187 (47) to form pHG1. Next, the 250 bp upstream of the *NEUT5L* mid-point (5′ *NEUT5L* homology) was amplified with a 5′ SphI site and 3′ overlap with the *C. albicans ENO1* promoter. The 850 bp directly upstream of the start of *ENO1* (p*ENO1*) was PCR-amplified with 5′ overlap to 5′ *NEUT5L* homology and 3′ overlap to GFP. *C. albicans* codon-optimized GFP with a downstream *C. albicans ADH1* terminator sequence (GFP-t*ADH1*) was PCR-amplified with 5′ overlap to p*ENO1* and a 3′ XhoI site. The 5′ *NEUT5L* homology, p*ENO1,* and GFP-t*ADH1* fragments were then joined by a second round of PCR. This 5′-SphI-5′*NEUT5L*-p*ENO1*-GFP-t*ADH1*-Xho1-3′ fragment was digested with SphI and XhoI and inserted into pHG1 digested with the same enzymes to form pHG2. The resulting plasmid was digested with SphI and AatII prior to transformation into *C. albicans*.

The live-cell imaging and RNA-seq experiments used fluorescent (time-lapse microscopy) or nonfluorescent (RNA-seq) white-opaque switching capable prototrophic MTL a/Δ derivatives of *C. albicans* SC5314 (an MTL a/α strain isolated from a patient with a *Candida* infection prior to 1968 [64–66]). SC5314 was converted to the MTL a/Δ mating type utilizing XmaI-digested pMBL745; loss of the alpha MTL was verified by colony PCR. The *SAT1* knock out cassette was then recycled by overnight growth in YEP media supplemented with 2% maltose, after which cells were plated on YPD plus 25 µg/mL nourseothricin for 24 h to identify small colonies that had lost the *SAT1* marker. These colonies were then replated on YPD plus 400 µg/mL nourseothricin to verify loss of the *SAT1* marker. MTL a/Δ status was then verified a second time by an additional round of colony PCR. Opaque cells of the resulting a/Δ strain were then isolated via plate-based white-to-opaque switching assays.

The fluorescent version of the MTL a/Δ SC5314 derivative strain used for the timelapse microscopy experiments constitutively expresses GFP from the *ENO1* promoter region (1,000 bp upstream of the *ENO1* gene) integrated at the Neu5L genomic locus constructed by transformation with SphI and AatII linearized pHG2. Integration of the GFP expression cassette was verified by colony PCR, and GFP expression was verified by detection of green fluorescence using the FL1-A (FITC) channel of an Accuri C6 Plus Flow Cytometer (BD Biosciences, 488 nm laser, 533/30 bandpass filter). The *SAT1* integration cassette was then recycled by overnight growth in YEP media supplemented with 2% maltose, after which cells were plated on YPD plus 25 µg/mL nourseothricin for 24 h to identify small colonies that had lost the *SAT1* marker. These colonies were then replated on YPD plus 400 µg/mL nourseothricin to verify loss of the *SAT1* marker. Integration of the GFP cassette was verified a second time by colony PCR and fluorescence detection, as described above. Opaque cells of the resulting a/Δ strain were then isolated via plate-based white-to-opaque switching assays.

### *C. albicans* culture conditions and co-incubation preparation

Unless otherwise noted, *C. albicans* strains were grown and assays were performed on synthetic complete defined media containing a yeast nitrogen base with 0.5% ammonium sulfate (6.7 g/L, BD #291940), amino acids (2 g/L), uridine (100 µg/mL), and 2% glucose (SCD +aa + Uri); 2% agar was added for plates. Growth was conducted in an ambient air incubator set to 25°C.

For both live-cell imaging and RNA-seq experiments, the *C. albicans* white and opaque strains were allowed to recover from glycerol stocks for 5 days on SCD +aa + Uri plates at 25°C. Between six and eight independent 5 mL overnight cultures were started for white and opaque cells from colonies that lacked detectable sectors of the other cell type. The following day, cell-type homogeneity was microscopically verified, after which the OD_600_ was determined, and each culture was used to seed two identical 5 mL overnight cultures at a starting OD_600_ of 0.002. The new overnight cultures were incubated for 21 h on a roller drum at 25°C so that they were undergoing logarithmic growth when they were collected to prepare for co-incubation with macrophages. Cell-type homogeneity was microscopically verified a second time at this point before proceeding with the wash steps.

The logarithmically growing *C. albicans* cells were collected via centrifugation (3,500 × *g*, 5 min) and then washed twice in phosphate-buffered saline (PBS: 137 mM NaCl, 2.7 mM KCl, 10 mM Na_2_HPO_4_, and 1.8 mM KH_2_PO_4_) containing 2.5 mM ethylenediaminetetraacetic acid (EDTA) and vortexed for 1 min after resuspension. The presence of EDTA and the extended vortexing are needed to ensure proper cell dissociation. After the final PBS-EDTA wash, cells were resuspended in 1 mL PBS (lacking EDTA). Concentrations of the final cell suspensions were determined via flow cytometry on an Accuri C6 Plus Flow Cytometer (BD Biosciences). Cell concentrations were normalized to 5 × 10^7^ cells/mL in PBS for use in subsequent macrophage co-incubation experiments.

### J774A.1 macrophage cell-line culture conditions and passaging

All macrophage experiments reported here utilized the J774A.1 cell line (ATCC, TIB-67). J774A.1 macrophages were expanded and maintained in 10 cm non-TC-treated Petri dishes in DMEM with 4.5 g/L glucose, L-glutamine, and sodium pyruvate (Corning 10-103-CV). This media was supplemented with 10% heat-inactivated FBS (Fischer Scientific, A5256801) and 1 × penicillin-streptomycin-glutamine (Gibco, 10378016). Macrophages were incubated at 37˚C with 5% CO_2_ and passaged to fresh DMEM at an initial concentration of 1–2 × 10^5^ cells/mL every 1–3 days. The macrophages were maintained in this way until use in subsequent experiments.

### Live cell imaging setup and image acquisition

Macrophages were seeded into each well of a 96-well ibidi black µ-Plate (ibidi, MSPP-89626) at a concentration of 5 × 10^5^ cells/mL in 300 µL DMEM and were incubated for 16–24 h at 37˚C with 5% CO_2_. Prior to infection with *C. albicans*, DMEM was replaced with pre-warmed (37˚C) HBSS (Life Technologies Corporation, 14025092) supplemented with 1 × penicillin-streptomycin-glutamine (Gibco, 10378016). Macrophages were incubated with HBSS for no longer than 1 h at 37˚C without CO_2_. During this time, *C. albicans* cultures were prepared for infection, as described above. Washed *C. albicans* strains were seeded into each well of the 96-well plate to achieve an MOI of 0.5 (white cells) or 1 (white and opaque cells). The 96-well plate was then incubated in an OkoLab Cage Incubator and the 96-well plate adapter for Nikon Ti2 (OkoLab, H201-NIKON-TI-S-ER) fitted with the 96-well plate magnetic holder (OkoLab, H201-MW-HOLDER) and the Koehler lid (OkoLab, H201-KOEHLER-LID) at 37˚C without CO_2_ for 30 min to allow phagocytosis to proceed. After 30 min, nonphagocytosed *C. albicans* cells were washed away by three gentle washes with warm (37˚C) HBSS. Following the wash steps, the HBSS media was replaced with DMEM supplemented with 10 µg/mL CFW (Thermo Fisher, R21507), staining *C. albicans* cells that were neither phagocytosed within 30 min nor removed by the HBSS washes. The plate was then incubated in the aforementioned OkoLab Cage Incubator setup at 37˚C with 5% CO_2_ for 24 h.

Images were captured using a Nikon Ti2-E microscope equipped with a Photometrics Prime 95B-25mm Camera and a Plan Apo 40 ×/0.95 DIC N2 objective lens. Nikon NIS Elements software was used to drive stage movement and acquisition across 16 (4 × 4) fields of view in each well of the 96-well plate; the Nikon Perfect Focus System was utilized. Images (DIC, FITC, and DAPI channels) were captured for each field every hour for 19–20 h. A temperature of 37°C and a 5% CO_2_ atmosphere were maintained using the aforementioned OkoLab Cage Incubator setup.

### Live-cell imaging image analysis

All images were scored manually by an unbiased analyst with no prior knowledge of the experimental setup. Before analyzing images, color and brightness were adjusted using the auto-scale adjustment in NIS Elements viewer. The analyst scored only macrophages containing either one or two *C. albicans* cells that were fluorescent green and not fluorescent blue in the initial time frame of the experiment (that is, *C. albicans* cells that had been phagocytosed by the macrophage in the first 30 min of the experiment). Macrophages that met these criteria were then tracked through each of the 20 time frames; macrophages that did not meet these criteria were not considered further. The analyst recorded the number of macrophages in each frame that met the initial criteria. When multiple macrophages in a given frame met the analysis criteria, they were scored independently from each other. For each macrophage that met the aforementioned criteria, the analyst recorded the number of *C. albicans* cells present at the initial time point, the time point at which the *C. albicans* cells first became blue (indicating escape from the macrophage), whether the *C. albicans* cells formed hyphae during the experiment, and whether the *C. albicans* cells became enlarged during the experiment. The data collected from each well and frame were then aggregated according to the cell type and MOI for further analysis and plotting. Time to escape values reported in the Results are for the MOI 1 data for each cell type and represent the time point at which at least half of the starting cells had been stained by CFW. Time to escape data for MOI 0.5 white cells, macrophages with only one *C. albicans* cell at t = 0, and macrophages with two *C. albicans* cells at t = 0 are presented in Fig. S1.

The aggregated data for this experiment are provided in File S1. A pdf file with 18 representative images from the time-lapse microscopy experiments, showing the frame from the 2- to 9-h time points for each image as well as representative moves, is available at Figshare through the DOI https://doi.org/10.6084/m9.figshare.30213922.

### Co-incubation RNA-seq setup

Macrophages were seeded into TC-treated 10 cm dishes with 10 mL DMEM at a concentration of 5 × 10^5^ cells/mL and incubated for 16–24 h at 37˚C with 5% CO_2_. Prior to infection with *C. albicans*, DMEM was replaced with pre-warmed (37˚C) HBSS, and the macrophages were incubated for no longer than 1 h at 37˚C without CO_2_ while *C. albicans* cultures were prepared for infection as described above. For the subsequent sample preparations, washed cells (*n* = 6) were divided to produce three distinct samples. For each replicate, the three distinct samples were seeded in the following order to minimize the time exposed to macrophages or media while also ensuring that wash and harvest steps were staggered appropriately for experimental feasibility. The three matched replicates that met the criteria after determining cell-type composition (see below) were subsequently processed and sequenced (see below).

#### *C. albicans* with macrophages for 2 h

Washed *C. albicans* strains were seeded into each 10 cm dish at an MOI of 1 (1 × 10^6^ cell/mL *C*. *albicans*). The plates were then incubated at 37˚C without CO_2_ for 30 min to allow phagocytosis to proceed. After 30 min, the nonphagocytosed *C. albicans* cells were removed by three gentle washes with warm HBSS. The HBSS was then replaced with DMEM, and the dishes were incubated at 37˚C with 5% CO_2_. After 2 h, the cells from each sample were then scraped, transferred to 15 mL Falcon tubes, and small aliquots collected from each sample for cell-type tracking (see below). Cells were then harvested by centrifugation (3,283 × *g*, 5 minutes), media was aspirated, and the pellets were then flash-frozen in liquid nitrogen and stored at −80˚C until subsequent RNA extraction and preparation.

#### *C. albicans* with macrophages for 0 h

For the 0 h controls, *C. albicans* were added to the 10 cm dishes with macrophages in DMEM to achieve a final *C. albicans* concentration of 1 × 10^6^ cell/mL. The *C. albicans* and macrophages were then immediately collected, sampled for cell-type tracking, and prepared for storage as described above. We note that these cells are exposed to macrophages and DMEM for 1 to 2 min during sample collection. This transient exposure to macrophages and/or DMEM during the seeding/ immediate harvesting step of the 0 h sample collection has the possibility of introducing transcriptional changes, and as such, the 0 h samples may differ from the baseline white- and opaque-transcriptional programs.

#### *C. albicans* without macrophages for 2 h

For the 2 h w/o MΦ controls, *C. albicans* were added at a concentration of 1 × 10^6^ cell/mL to 10 cm dishes in 10 mL DMEM that lacked macrophages. The *C. albicans* cells were then incubated at 37˚C with 5% CO_2_ for 2 h, before being collected, sampled for cell-type tracking, and prepared for storage, as described above. We note that the 2 h w/o MΦ controls lack the HBSS incubation and wash steps that were present in the 2 h MΦ samples because these steps, intended to remove unphagocytosed cells, would have washed away the *C. albicans* cells which were, by design, not phagocytosed. Although it is possible that this may introduce transcriptional differences between the 2 h MΦ and the 2 h w/o MΦ samples, as noted in the results, our data largely recapitulate the data from the *C. albicans* a/α white cell “macrophage-enriched” set previously reported by Pountain and colleagues (41).

### Determining the cell-type composition of RNA-seq samples

To determine the cell-type composition of all *C. albicans* samples, small aliquots were collected from each sample during the harvesting process. These aliquots were diluted 1:100 with dH_2_O, and 100 µL was plated on each of two SCD + aa + Uri plates. These plates were then incubated for 3 days at 25°C, after which the plates were manually counted and scored. Two phenotypes were scored: (i) the total number of colonies and (ii) the number of colonies not of the original cell type (e.g., white colonies for a sample whose starting culture was of opaque cells). The switching frequency was calculated as 100 * (number colonies not of the original cell type) / (total number of colonies); switching frequencies are provided in Table S1.

### RNA extraction, library preparation, and sequencing

Three matched replicates for each condition (0 h, 2 h MΦ, 2 h w/o MΦ) were chosen by considering switching frequencies in all conditions. Samples with the lowest switching frequencies across all three conditions were selected for RNA extraction and library preparation. Frozen cell pellets were thawed on ice. To each pellet, 1 mL TRIzol (ThermoFisher 15596026) was added, and tubes were vortexed vigorously until the pellets were completely suspended. Tubes were incubated at room temperature for 5–10 min with occasional vortexing and back-pipetting until samples became less viscous. Samples were then centrifuged at 6,000 × *g* for 5 min to pellet the unlysed *C. albicans* cells. The supernatant, which contained the lysed macrophage cells, was discarded. RNA was extracted from the *C. albicans* cell pellets using the Ambion RiboPure Yeast Kit (Invitrogen AM1926) following the manufacturer’s protocol including a DNase I treatment step. The RNA concentration was measured using a NanoDrop 2000C (Thermo Scientific), and RNA integrity was measured using a TapeStation 4200 (Agilent) and the TapeStation High-Sensitivity RNA kit (Agilent 5067-5579, 5067-5581, and 5067-5580). All RNA samples had RIN scores greater than 7.

Libraries were prepared from 125 ng of input RNA using the Lexogen Quant-Seq FWD Kit (Lexogen**,** 191.96) with the following modifications to the manufacturer’s protocol. Libraries were amplified with indexing primers using the fluorescent amplification of next-generation sequencing libraries technique (67). Briefly, the 2.5× SYBR solution provided with the Lexogen PCR Add-on and Reamplification Kit V2 (Lexogen, 208.96) was added to the endpoint PCR mix provided with the Lexogen Quant-Seq FWD Kit at a concentration of 0.035% (vol/vol). Additionally, the reaction volumes were reduced by half relative to the recommended volumes in order to accommodate fluorescence detection in a 384-plate format. Amplification was monitored by fluorescence detection using the CFX384 Touch Real-Time PCR System (Biorad). Pooling volumes were calculated by normalizing the end-point values to a maximum volume of 2,000 nL, and samples were pooled using an Echo 525 High Volume Transfer Liquid Handler (Beckman) as described by Chiniquy and colleagues (67). Following pooling, the final library was purified using the protocol for purification following end-point PCR from the Lexogen Quant-Seq FWD Kit. The resulting pooled library was quantified using a Qubit 2.0 Fluorometer (Invitrogen) via the Qubit dsDNA (HS) High-Sensitivity Assay Kit (ThermoFisher, Q32851) and assessed for quality with a TapeStation 4200 (Agilent) using the TapeStation High-Sensitivity DNA kit (Agilent, 5067-5585, 5067-5587, and 5067-5584). Finally, the libraries were diluted to a concentration between 5 and 10 nM and sequenced (100 bp, single-end reads) on a NovaSeq X Plus (Illumina) at the University of California, San Francisco’s Center for Advanced Technology. Each sample yielded ~ 15 million reads.

### RNA-Seq data processing

RNA-seq data were analyzed following the protocol outlined in Gause et al. (68). Briefly, fastq files were filtered for quality and trimmed of Illumina sequencing adapters and poly(A) tails using Fastp (0.20.1) (69). FastQC (0.11.9) was used to perform quality checks of data (70). Reads were mapped using STAR (2.7.9 a) (71) to the *C. albicans* SC5314 Assembly 22 (version A22-s07-m01-r105) chromosome file from the *Candida* Genome Database (CGD) (72), modified to contain only A allele chromosomes. To generate a gene annotation file from reverse transcript data derived from the poly(A) priming technique, bam files were converted into bedgraph files with a bin size of 20 base pairs using Deeptools (3.5.3) (73) and were analyzed for 3′ peaks using Peakstream with default parameters (68). Read counts were extracted from each bam file using FeatureCounts from the Subread package (2.0.1) (74) with the annotation file (GTF format) generated by Peakstream and the gene annotation for *C. albicans* SC5314 Assembly 22 (version A22-s07-m01-r105) from CGD (72) modified to contain nTAR fragment annotation (24). Genes with fewer than 10 reads across all samples were excluded from further analysis. PyDESeq2 (version 0.4.11) was used for differential expression analysis in Python. Normalization of raw counts was performed using the default size factor method in PyDESeq2. Differential expression for all relevant comparisons was modeled using the PyDESeq2 implementation of the DESeq2 statistical methods (75). The normalized read counts for the macrophage co-incubation experiment are provided in File S2. A PCA plot for each of the samples for each of the repeats is provided in Fig. S2A.

### Analyzing differentially expressed genes

All gene names and gene annotations used in this paper are taken from the *C. albicans* SC5314 Assembly 22 Current Chromosomal Feature file downloaded from CGD on 14 August 2024. The analyses presented in the text focus solely on ORFs; neither nTARs nor hypothetical features are included in gene counts or statistical analyses. The following statistical thresholds were applied: genes with at least fourfold log2 fold changes (L2FC) between conditions were required to have a P_adj_ less than or equal to 0.05; genes in the indicated L2FC range that did not meet the required P_adj_ threshold had their L2FC values set to 0. No statistical threshold requirements were applied to the *C. albicans* a/α white cell macrophage-enriched gene set reported by Pountain and colleagues (41). Statistical analysis of the overlap between sets of genes was performed using the hypergeometric distribution (“HYPGEOM.DIST”) function in MS Excel with the cumulative distribution function (“Cumulative”) option set to “TRUE”; subtracting this value from 1 gives the probability of obtaining more than that number of genes in a population (e.g., the degree to which the first set of genes is over-represented in the second set of genes). We note that MS Excel returns a value of “0” for results less than 1e-15; as such, we report those results as “< 1e-15.” The transformed RNA-seq data for the macrophage co-incubation experiment, both with and without the aforementioned statistical thresholds applied, are compiled in File S2.

In order to verify that the decrease in the number of white- and opaque-enriched genes between the 0 h and the 2 h MΦ samples was not an artifact resulting from our default thresholds (at least a fourfold change between samples, P_adj_ less than 0.05), we repeated this comparison for three, six, and eightfold change thresholds with P_adj_ < 0.05 as well as for two, three, four, six, and eightfold thresholds with P_adj_ < 0.01. These comparisons are provided in the “Alternative Thresholds” tab of 2. Other thresholds for this can be evaluated using the “Differential Expression” tab in File S2.

Biological process, molecular function, and cellular component GO term enrichment analyses were performed using the PANTHER database’s statistical overrepresentation test with *Candida albicans* selected for both the organism and default whole-genome lists and choosing the Fisher’s Exact and Calculate False Discovery Rate options (https://www.pantherdb.org/) (76, 77).

KEGG Pathway analysis was conducted using a custom Excel file built around a list of the subset of 5,998 *C*. *albicans* genes with a valid KEGG identity that were associated with each of 121 KEGG pathways downloaded from the KEGG website on 13 January 2023 (*Candida albicans* SC5314, Organism T00189, https://www.genome.jp/kegg-bin/show_organism?org=T00189) (78, 79). In brief, the file compares how many genes from a query gene set (e.g., opaque cell “macrophage-enriched”) map to each KEGG pathway relative to how many of the genes from the 5998 gene set map to the same KEGG pathways and then uses the hypergeometric distribution function in Excel to determine the probability of having at most the number of genes that map to the pathway and the probability of having at least one more than the number of genes that mapped to the pathway. If we consider the 108 KEGG pathway maps that are associated with at least 5 of the 5998 *C*. *albicans* genes, the Bonferroni Correction gives a statistical threshold of 4.6e-4 for an α of 0.05. The custom Excel file for KEGG Pathway analysis is provided as File S4.

## Data Availability

The raw RNA-seq data from the *C. albicans*–macrophage co-incubation experiment are available at NCBI as BioProject number PRJNA1321335. The normalized read counts and transformed RNA-seq data for the macrophage co-incubation experiment, both with and without statistical thresholds applied, are provided in File S2. The aggregated data for the time-lapse microscopy experiments are provided in File S1. A PDF file with 18 representative images from the time-lapse microscopy experiments, showing the frame from the 2- to 9-h time points for each image, as well as representative movies, is available at Figshare through the DOI https://doi.org/10.6084/m9.figshare.30213922.

## SUPPLEMENTAL MATERIAL

File S1 msphere.00690-25-s0001.xlsx Compiled data for the time-lapse microscopy experiments.

File S2 msphere.00690-25-s0002.xlsx Processed, transformed RNA-seq data for the macrophage co-incubation experiment.

File S3 msphere.00690-25-s0003.xlsx Oligonucleotides, plasmids, and strains used in this study.

File S4 msphere.00690-25-s0004.xlsx KEGG pathway analysis built using the subset of 5,998 *C. albicans* genes with a valid KEGG identity that were associated with each of 121 KEGG pathways.

Figure S1 msphere.00690-25-s0005.tif White and opaque cell proliferation differences following phagocytosis by macrophages are not affected by the MOI or the number of cells in the macrophage at *t* = 0 h.

Figure S2 msphere.00690-25-s0006.tif Principal component analysis of the co-incubation RNA-seq experiment and heatmap of the 133 genes that are at least fourfold macrophage enriched in white cells.

Figure S3 msphere.00690-25-s0007.tif In both white and opaque cells, the "macrophage-enriched" response to phagocytosis involves a mixture of upregulating genes in macrophages and downregulating genes when macrophages are not present.

Figure S4 msphere.00690-25-s0008.tif Gains and losses of opaque and white cell type-enriched genes between the 0 and 2 h MΦ samples are driven by a mixture of up- and downregulation in the two cell types.

Figure S5 msphere.00690-25-s0009.tif The opaque cell type-enriched gene sets significantly overlap with the macrophage-enriched gene sets from both white and opaque cells.

Table S1 msphere.00690-25-s0010.xlsx Cell type switching rates for white and opaque samples.

ASM does not own the copyrights to Supplemental Material that may be linked to, or accessed through, an article. The authors have granted ASM a non-exclusive, world-wide license to publish the Supplemental Material files. Please contact the corresponding author directly for reuse.
