# Supplementary figures and images for: Response of Candida albicans white and opaque cells to phagocytosis by macrophages suggests that opaque cells are “pre-adapted”
Source: mSphere. 2025 Dec 18;11(1):e00690-25. doi: 10.1128/msphere.00690-25 (PMC12838362; doi:10.1128/msphere.00690-25)

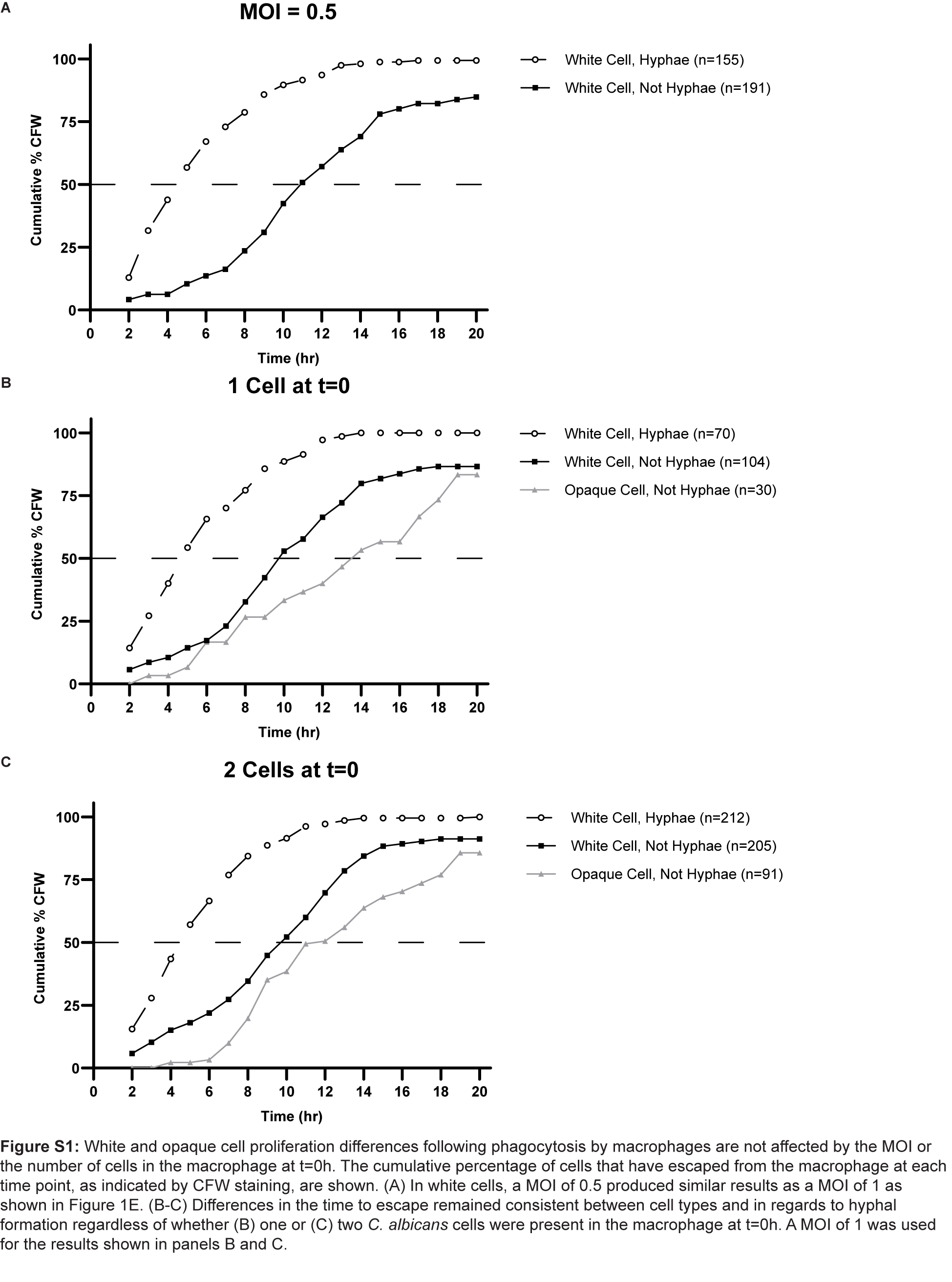

Supplement: Figure S1 — White and opaque cell proliferation differences following phagocytosis by macrophages are not affected by the MOI or the number of cells in the macrophage at t = 0 h. [file msphere.00690-25-s0005.tif]

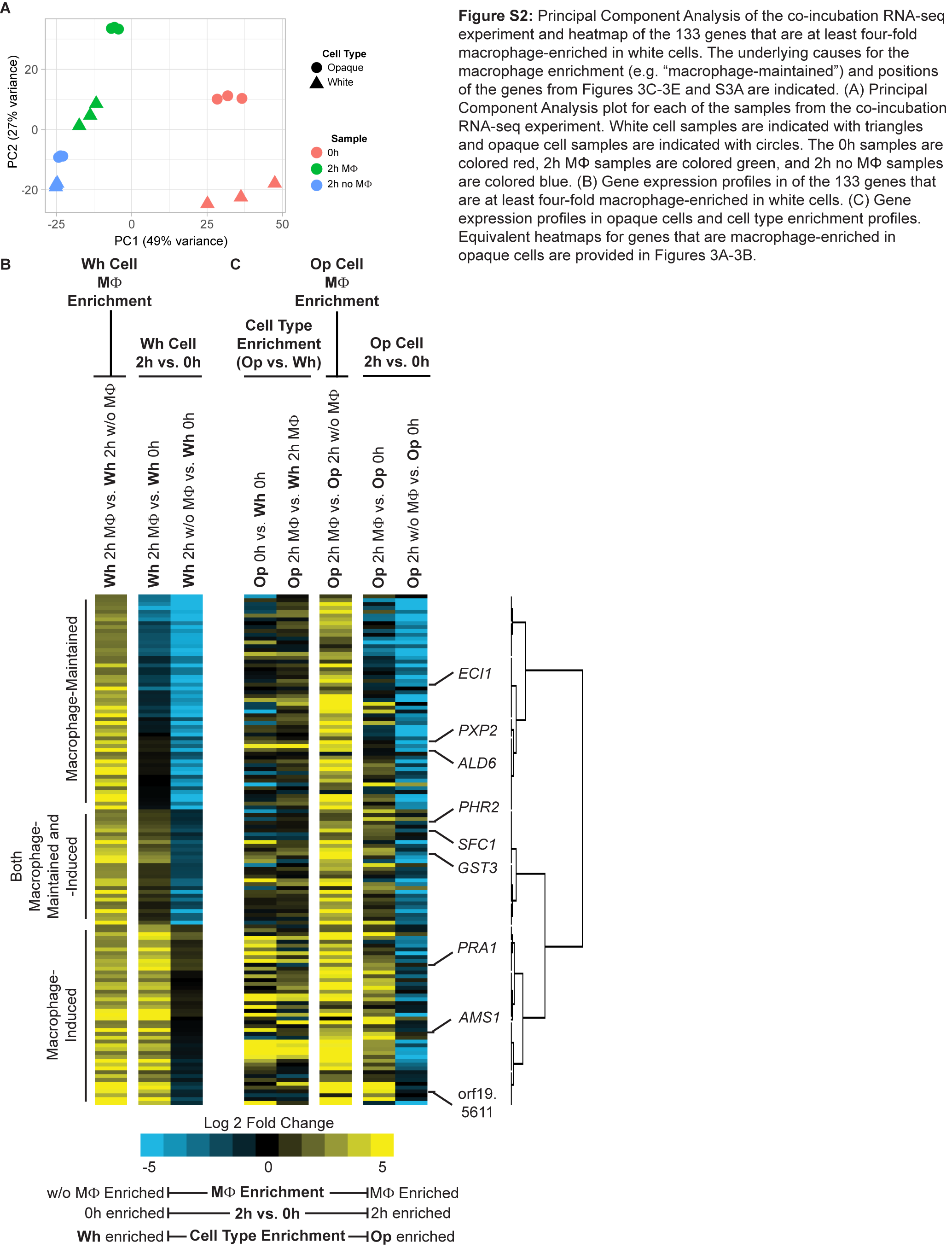

Supplement: Figure S2 — Principal component analysis of the co-incubation RNA-seq experiment and heatmap of the 133 genes that are at least fourfold macrophage enriched in white cells. [file msphere.00690-25-s0006.tif]

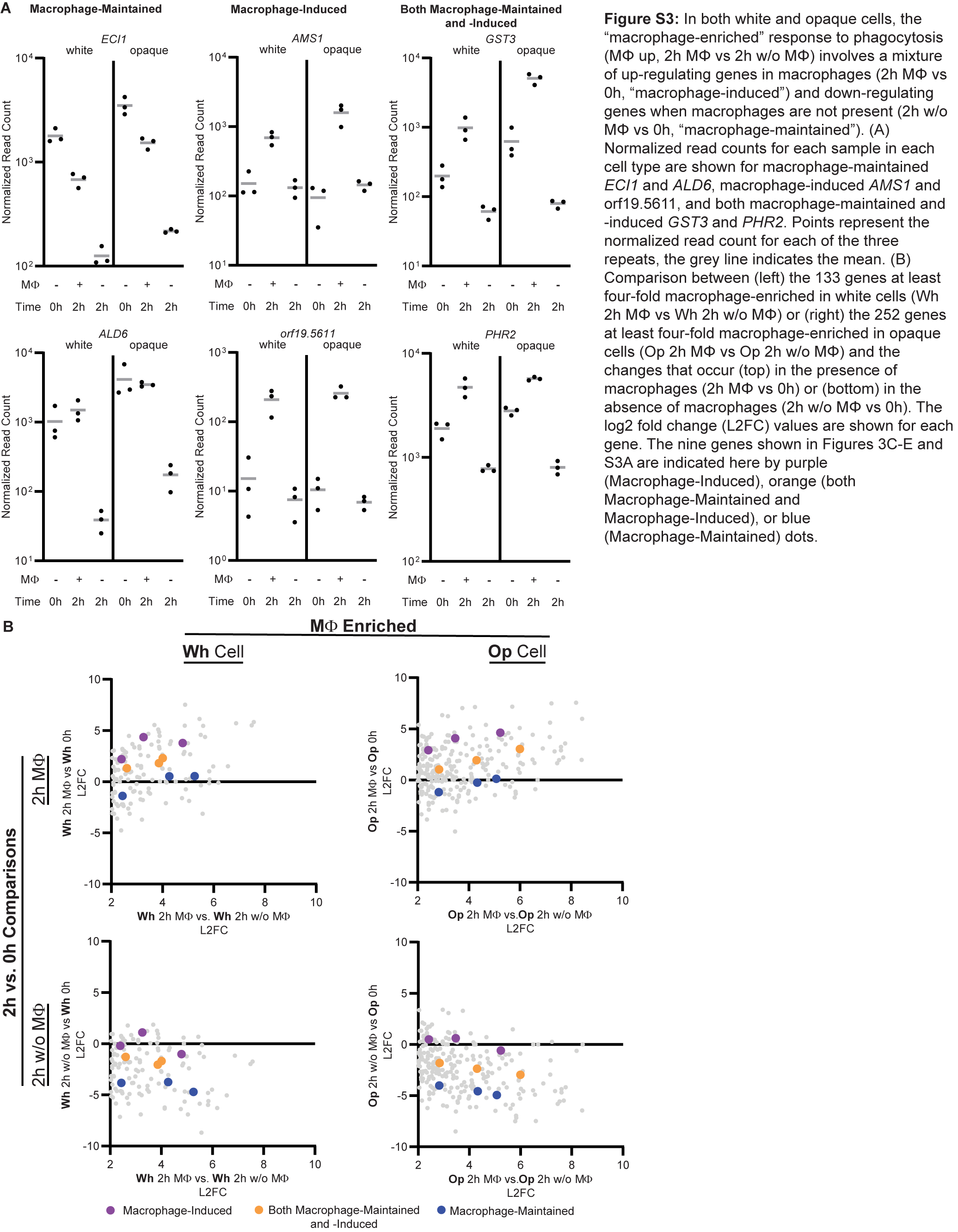

Supplement: Figure S3 — In both white and opaque cells, the "macrophage-enriched" response to phagocytosis involves a mixture of upregulating genes in macrophages and downregulating genes when macrophages are not present. [file msphere.00690-25-s0007.tif]

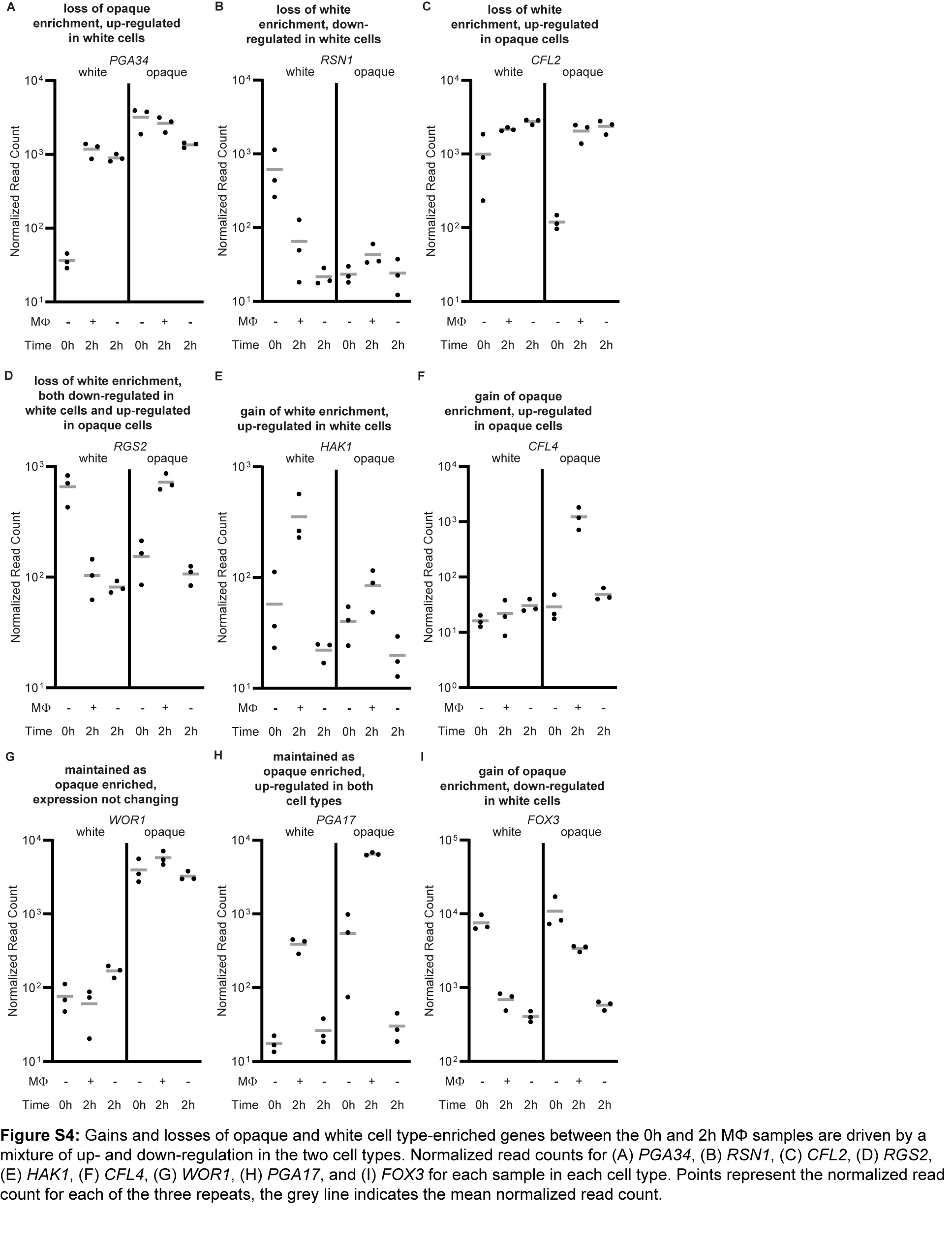

Supplement: Figure S4 — Gains and losses of opaque and white cell type-enriched genes between the 0 and 2 h MΦ samples are driven by a mixture of up- and downregulation in the two cell types. [file msphere.00690-25-s0008.tif]

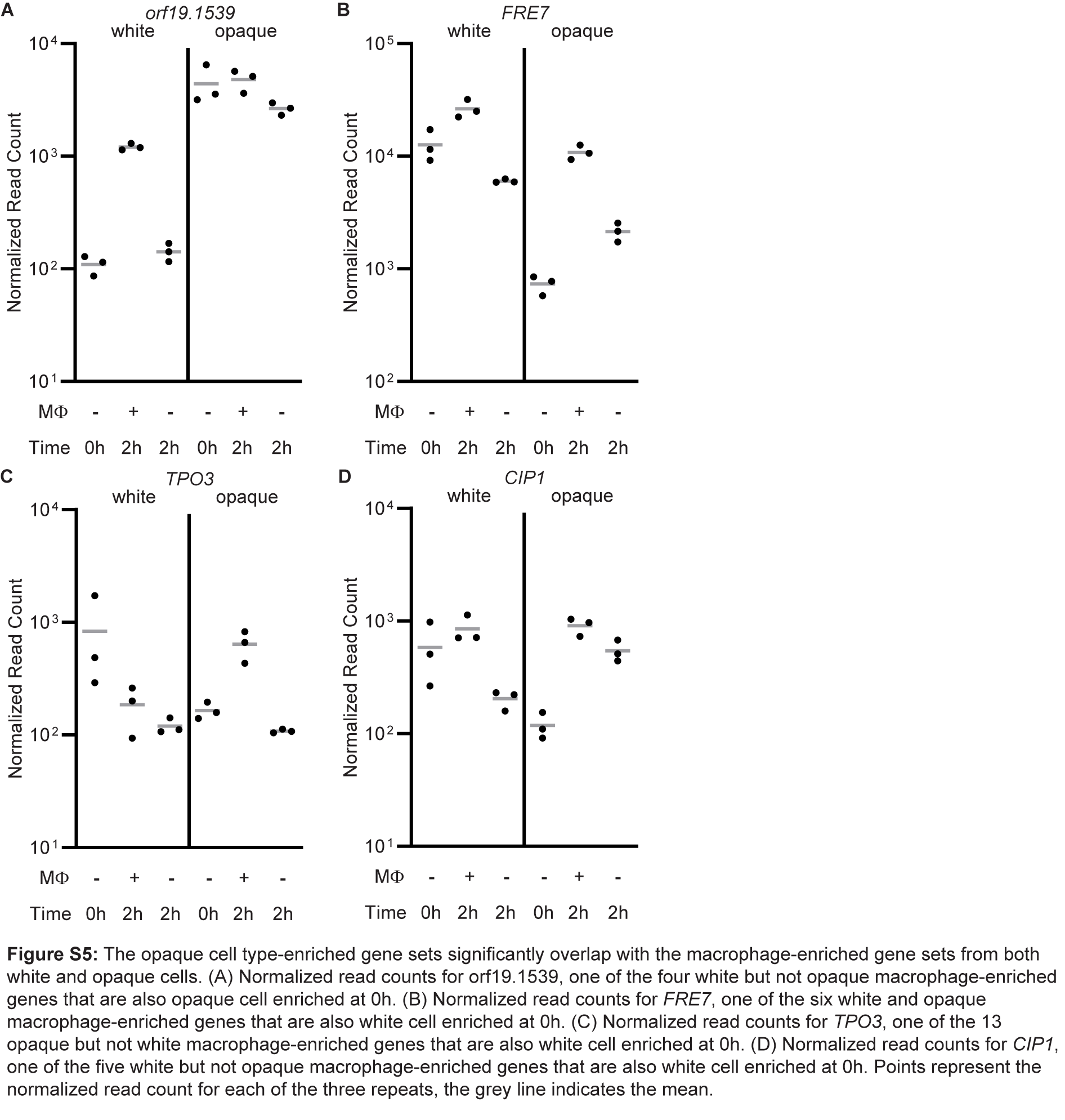

Supplement: Figure S5 — The opaque cell type-enriched gene sets significantly overlap with the macrophage-enriched gene sets from both white and opaque cells. [file msphere.00690-25-s0009.tif]
